# Enhancing the robustness of recommender systems against spammers

**DOI:** 10.1371/journal.pone.0206458

**Published:** 2018-11-01

**Authors:** Chengjun Zhang, Jin Liu, Yanzhen Qu, Tianqi Han, Xujun Ge, An Zeng

**Affiliations:** 1 School of computer and software, Nanjing University of Information Science and Technology, Nanjing 210044, P.R. China; 2 ShuKun (BeiJing) Network Technology Co., Limited, Room 313, Building 3, No. 11, Chuangxin Road, Science Park, Changping District, Beijing, China; 3 Jiangsu Collaborative Innovation Center of Atmospheric Environment and Equipment Technology (CICAEET), Nanjing University of Information Science and Technology, Nanjing 210044, P.R. China; 4 School of Computer Science and Technology, Colorado Technical University, Colorado Springs, 80907, United States of America; 5 School of Systems Science, Beijing Normal University, Beijing, 100875, P. R. China; Victoria University, AUSTRALIA

## Abstract

The accuracy and diversity of recommendation algorithms have always been the research hotspot of recommender systems. A good recommender system should not only have high accuracy and diversity, but also have adequate robustness against spammer attacks. However, the issue of recommendation robustness has received relatively little attention in the literature. In this paper, we systematically study the influences of different spammer behaviors on the recommendation results in various recommendation algorithms. We further propose an improved algorithm by incorporating the inner-similarity of user’s purchased items in the classic KNN approach. The new algorithm effectively enhances the robustness against spammer attacks and thus outperforms traditional algorithms in recommendation accuracy and diversity when spammers exist in the online commercial systems.

## Introduction

The emergence and popularization of the Internet have brought tremendous information to individuals, leading to serious information overload problems [1–4]. In addition to this, some merchants hire the network water army to add false purchase records or praise to their products in order to increase the sales of their own goods. Obviously, the spamming behavior from these Internet marketers will make the information overload problem even worse, as users are more likely to be misled by these spammers and waste their valuable time and money on products that they are actually not interested in.

A promising way to solve the problem of information overload is through recommender systems [5–8], which recommend information and products to users according to their previous behavior records. Compared with search engines, recommender systems make predictions based on the analysis of users’ interest preferences [9, 10]. Once users’ preferences are extracted by the recommender systems, a small set of relevant products will be recommended to users. In order to work appropriately in different circumstances, numerous different algorithms have been developed, such as content-based analysis [11–13], spectral analysis and iterative self-consistent refinement, which are mainly used to filter irrelevant information. Some algorithms based on physical principles are also used to design recommendation algorithms, such as the mass diffusion [14–17] and conduction process [18–20]. By combining the mass diffusion and heat conduction processes [21, 22], one can get a hybrid algorithm [23–25] which is shown to be superior to the original two algorithms in both recommendation accuracy [26–29] and recommendation diversity [30, 31].

Many current mainstream recommendation algorithms are based on user similarities [32]. Two users are assigned with high similarity if they have chosen a lot of common items. The success of these kind of methods is because individuals who have purchased many common items are more likely to share the same preference in the future. However, in the real networks, there will inevitably be some fictitious and redundant information which can affect the precision of recommender systems [27, 33]. For example, in the e-commerce websites there are some irresponsible users who tend to choose at random in the system, thereby misleading recommender systems [28, 34, 35]. A even worse situation is that on the Internet, some companies may employ “Internet water army” to publish false information so as to misguide users. For example, the Internet water army may be hired to publish favorable comments for a lousy movie, thus misleading recommender systems to mistakenly suppose the film to be popular and recommend it to real users. These problems are increasingly rampant in many e-commerce sites, which have caused a significant challenge to the recommender systems.

Actually, over the past few decades, the users’ comment information on items or services have provided important reference information for other users in the social network. However, with a large number of spammers mixing into the system, a great deal of false information is published which largely mislead users [36, 37]. Numerous detection methods have also been developed to identify these spammers from the system. By using reviewers’ behaviors, text similarity and rating patterns, these methods are designed to identify the spammers from the online systems [38].

In email systems, there are two kinds of methods for identifying and filtering of spammers: one is to maintain a white list and blacklist for each user, and identify spammers by identifying the identity of the mail sender [5, 16, 21]. The other is to analyze the content of email, and filter spammers through the keywords in the email. There is also a spammer filtering technology based on DNS, which filters spammers by maintaining a list of IP addresses that have been identified as spammers [5, 16, 21].

In online commercial and social networks, there are also numerous methods to detect spamming behaviors. Zhou et al. put forward a correlation-based reputation algorithm to cope with the spammers of web-based rating systems [39, 40]. In this algorithm, the popularity of each user is adjusted by this user’s rating vector and the correlation coefficient of the weighted average rating of the corresponding item. Zhu et al. proposed a SMFSR method to identify spammers in social networks based on the user’s social relationships and social behaviors [41]. Benevenuto et al. used the classification strategy to identify the garbage users amaong video users in social networks by customizing the attributes and social characteristics of video users [42]. Las-Casas et al. proposed a method called SpaDeS to identify spammers in source network [43]. This method relies on the supervised classification technique, and only works according to network-level metrics. Therefore, it does not need the specific content of the information. The company Facebook developed proposed a EdgeRank algorithm to identify spammers, which scores for this post according to some attributes of each post. The post of low score is more likely to be spammering behaviors [36, 42, 43]. In addition to these works, spamming behaviors are studied from the pespective of semantic analysis. Gonzalez et al. proposed a method based on the Natural Language Processing, analyzing and classifying the sentiment according to the entities mentioned in each tweet [44]. Gil et al. systemically studied the developments of the legal system regarding knowledge sharing models among individuals, which had been significantly changed by the advent of social networks [45]. Moch*ó*n studied how to detect risk of propagation and market behavior based on analyzing relations between the financial market participants [46]. J. F. L*ó*pez-Quintero designed a functional architecture based on an algorithm of machine learning which integrated a semantic analysis algorithm with the Web 2.0 application from unstructured information [47].

Although there have been a huge amount of research on spammers in complex networks, there is still a lack of systematic research on the effects of spammers on recommender systems. This paper aims to study systematically effect of spamming behaviors on recommender systems. By adding virtual users to the system, we can simulate different types of spamming behaviors. We focus on how the spammers’ behaviors affects the performance of recommender systems, including recommendation accuracy and diversity. Eventually, we propose an effective algorithms which successfully avoid using the unreliable information and maintain high recommendation accuracy when spammers exist.

## Methods

### Data description

In this paper, the datasets that we will use are the subsets of data obtained from online systems: Amazon(http://www.Amazon.com), RYM(http://rateyourmusic.com) and Delicious(https://del.icio.us). Each data can be represented by a bipartite network consisting of users and items. The links between a user and an item indicates that the user has selected the item before. The descriptions of these datasets are given in Table 1. Throughout this paper, we will main present the results with the Amazon data. The results of RYM and Delicious are shown in the Supplementary Information (SI). All data was collected according to Amazon, RYM and Delicious’s terms of service and privacy conditions.

**Table 1:** The basic statistics of the empirical data.

network	Users	Items	Links
Amazon	2988	53770	66563
Delicious	1000	76179	126369
RYM	3378	4489	66408

### Recommendation algorithms

In order to study the effect of spammers on the recommender systems, we compare the accuracy and diversity metrics of recommendation algorithms in real networks and networks with different proportions of spammers. We mainly consider two kinds of recommendation algorithms: mass diffusion (MD) [15–17] and collaborative filtering (CF) [18–20]. The reason why we choose these two methods is that MD method is a recommendation algorithms based on the mass diffusion process which has a profound influence in the physic community whereas CF is a algorithms which has been widely applied in many e-Commerce Systems including Amazon, Facebook and Twitter. We focus on how the recommendation performance is influenced when we gradually add spammers to the user-item bipartite networks.

The MD method [15–17] is applied on the user-item bipartite network with *N* users and *M* items. The bipartite network can be expressed with an adjacency matrix *A*. If a user *i* has selected an item *α*, we denote the element *a*_*iα*_ = 1 in the adjacency matrix, otherwise *a*_*iα*_ = 0. As MD is a personalized recommendation algorithm. It needs to be applied to each user. For a user *i*, the first step is to assign a unit of resources to each item selected by user *i*. Then these resources are distributed in this bipartite network. We use a vector *f*^*i*^ to record the initial resources on all items. That is to say that the resources obtained by the item *α* can be expressed as $f_{i}^{\alpha }$. In order to conduct recommendation for user *i*, we set each element $f_{i}^{\alpha }=a_{i\alpha }$ of the vector *f*^*i*^. Then, the propagation process starting from user *i* can be expressed as ${\tilde{f}}_{i}=Wf_{i}$ where *W* is the diffusion matrix with each element computed as

$$W_{\alpha \beta }=\frac{1}{k_{\beta }}\sum_{l=1}^{N}\frac{\alpha_{l\alpha }\alpha_{l\beta }}{k_{l}}.$$ (1)

Here, *k*_*β*_ is the degree of item *β*, and *k*_*l*_ is the degree of user *l*. The final recommendation score for each item is equal to its received resource score in this diffusion process.

The CF algorithm [5, 6, 9, 10] makes recommendation based on the similarity of users and items. In this paper, we consider the user-based CF which relies on user similarity for recommendation. In order to recommend items to a target user *i*, the algorithm first calculate the topological similarity *s*_*ij*_ between user *i* and any other user *j*. Finally, the recommendation score for each item *α* to user *i* can be expressed as

$$\tilde{f_{i}^{\alpha }}=\sum_{j=1}^{N}s_{ij}a_{j\alpha }.$$ (2)

Here we use the Jaccard Index [6] to compute the similarity.

### Metrics

In order to measure the accuracy of an recommendation algorithm, the links in real data has to be randomly divided into two sets: training set *E*_*T*_ and probe set *E*_*P*_. The recommendation algorithms use the information of training set to generate recommendation list. The probe set is used to compare with the recommendation list to finally measure the recommendation accuracy. Usually *E*_*T*_ takes up 90% links of the whole data set, and *E*_*P*_ consists of the rest 10% links.

A common index for measuring the recommendation accuracy is the ranking score. The ranking score metric is computed on each individual user. For a target user, we first focus on the items that have not been selected by him/her, and then generate the ranking list of these items based on these items’ recommendation score in descending order. Then for each of the user’s selected items in the probe set, we need to calculate the ranking of the item among this ranked list. For example, the recommendation list length of the user is *L*, and the ranking of the probe set item is *a*, then the ranking score of the item for this user is *a*/*L*. We need to calculate the average value of all probe set items’ ranking score. The expression of the ranking score is as follows:

$$RS=\frac{1}{|E_{P}|}\sum_{i\alpha \in E_{P}}RS_{i\alpha }.$$ (3)

where *iα* denotes the probe link connecting user i and item *α*. According to the definition of the ranking score, the higher recommendation accuracy is, the lower is the value of the ranking score is.

Another metrics for accuracy is the Precision index. Different from the ranking score metric, precision computes accuracy with only top-*L* items in the recommendation list. Assuming *m*′ items in a target user’s recommendation list are his/her probe set items, the precision of this user can be expressed as

$$P=\frac{m^{\prime }}{L}.$$ (4)

The precision of the whole system can be obtained by averaging *P* of all individual users.

Diversity is another important issue in recommendation. The diversity metric is to calculate the Hamming distance among items in the recommendation list (top-*L* items) for different users. Assuming there are *L*′ common items in the recommendation lists of user *i* and user *j*, then the Hamming distance between random these two users can be computed as

$$H=1-\frac{L^{\prime }}{L}.$$ (5)

Obviously, the larger Hamming distance is, the higher is the recommendation diversity. The diversity of the whole system is obtained by averaging *H* over all user pairs.

In addition, we also consider a metric called novelty, which calculates the average degree of the top-*L* items in the recommendation list of users. Mathematically, the metric can be represented as:

$$N=\frac{1}{UL}\sum_{i=1}^{L}\sum_{\alpha \in O_{i}^{L}}^{}k_{\alpha }.$$ (6)

where *U* is the number of users in the system and $O_{i}^{L}$ denotes the set of items in top *L* places of user *i*’s list. Obviously, a smaller value in the novelty metrics indicates that the item recommended by recommender systems is more unpopular, otherwise more popular.

## 1 Results

**Fig 1:**
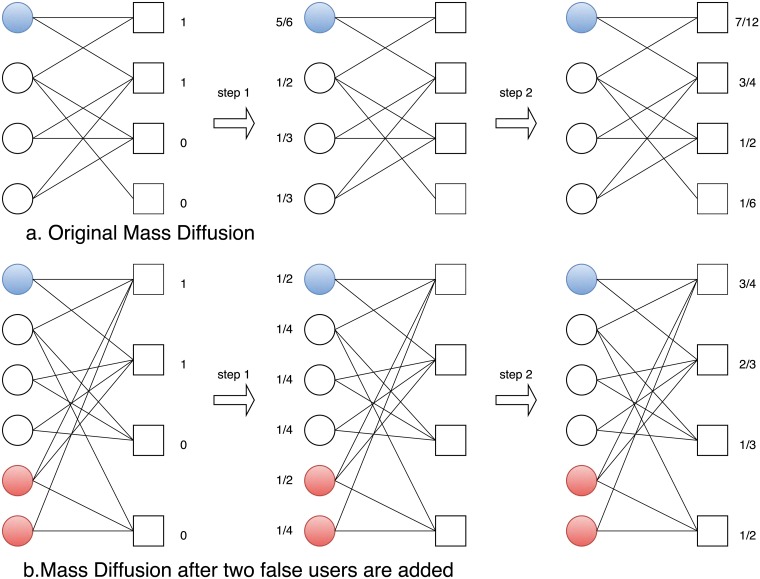
(Color online) (a) Mass diffusion on the original network and (b) Mass diffusion on the network where two spammers are added to the network.

To begin our analysis, the links created by spammers may largely distort the user/item similarities in collaborative filtering algorithms and the received resource in mass diffusion algorithms, resulting in a significant change in the recommendation list for each user. This eventually decreases the recommendation accuracy. An example for MD recommendation algorithm is illustrated in Fig 1. As we can see, Fig 1(a) shows the two-step diffusion process when MD recommends items for the target user (in blue). Fig 1(b) shows also the same process when two spammers are added into the network. Obviously, the final received resources of items are changed after two spammers are added. In order to simulate the behavior of spammers on the user-item bipartite network, we let each spamming user connect a certain number of edges in the real network according to the following strategies. In the following, we will also explain them how these strategies influence recommender systems respectively.

- **Each spamming user connects randomly to items**. This strategy has very limited influence on recommender systems, because it has the equal influence on all items. Since false connections are randomly added to all items, hot items remain hot and unpopular items remain low-frequent. Furthermore, this strategy makes no sense for the Internet water army because it does not meet the water army’s purpose.
- **Each spamming user only connects to the smallest degree items**. Actually, this strategy used to be widely adopted by the Internet water army as it can rapidly increase the degree of niche items and it does work on popularity-based recommendation algorithms. However, it fails to build relationships with other real users, as all spamming users are well-connected with each other by their selected niche items while most recommendation algorithms work based on the relationship between users and items, thus, it has little impact on recommender systems.
- **Some edges are connected to the smallest degree items, and the rest edges are connected to the largest degree items**. Actually, this strategy has relatively higher influence on the system than the first two strategies when a large number of spamming users are added to the system. However, the influence is very limited. This is due to the fact that among users who have bought the most popular items (high degree), this strategy will successfully increase the recommendation score of unpopular items (cold items) in their (people who have bought the most popular items) recommendation list. This is why recommendation accuracy is slightly decreased as some cold items are pushed to the recommendation list of some users. However, there are so many users who have bought hot items and among most of these users, these niche items are on the bottom of the recommendation list, although this strategy can slightly increase the score of niche items, their score still cannot be high enough to enter the recommendation list, thus, the recommendation accuracy is not so influenced.
- **Some edges are connected to the largest degree items, and the rest edges are connected randomly to items**. Actually, this strategy has the most significant influence on the system. This is because this strategy will further increase the recommendation score of hot items, the consequence is that more hot items will be pushed to the recommendation list of users. In other words, this strategy will make the recommendation lists of most users full of hot items. However, in really world, not all users tend to purchase hot items, therefore, the recommendation accuracy sharply decreases. In our paper, we did not analyze this strategy for the simple reason that it makes no sense to push popular items to users as the Internet water army is only interested in pushing cold items.
- **Some edges are connected to the smallest degree items, and the rest edges are connected randomly to items**. This strategy meets the requirements of the Internet water army. This is because, on one hand, it builds relationship and similarity with normal users by its random connection to items. On the other hand, by selecting cold items, it gives a higher recommend score to cold items so they are more likely to be recommended to normal users.

As we discussed above, in simulation, we also found that the spammers with the first three strategies have limited influence on the recommendation accuracy. In other words, the MD and CF recommendation algorithms are robust to these three spamming behaviors. We then compare strategy 4 and strategy 5, and found that the recommendation accuracy generally decreases faster in strategy 4. Our results imply that if the Internet water army wants to attack the recommender systems, recommender systems will be significantly influenced when one part of the edges connect to the hottest items, and the other part of the edges connect to items randomly.

**Fig 2:**
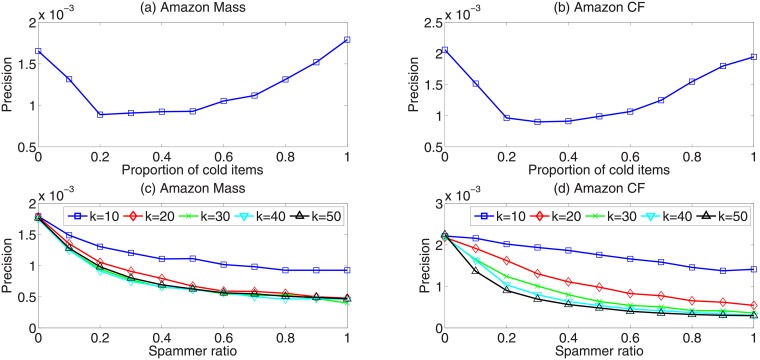
(Color online) (a), (b) show the relationship between recommendation accuracy and the ratio of the number of links to cold items and the number of total links. (c), (d) show the relationship between recommendation accuracy and the number of links of every false user.

Considering that the Internet water army is usually employed by some water army companies to promote niche items (small degree items) in online commercial systems, we mainly focus on strategy 5 in the following analysis. Specifically, we add 20% spammers to the real networks and let their number of links equal to the average user degree in the original network. We investigate how the fraction of connected cold items and fraction of randomly selected items influence the recommendation accuracy. As shown in Fig 2(a) and 2(b), the horizontal axis is the fraction of links connected to cold items (the remaining links are randomly linked to items), and the vertical axis represents recommendation accuracy measured by precision. It can be easily observed that when the fraction of cold items in the total edges is around 20%, the recommendation accuracy is affected most significantly.

We further study how the number of spammers affects the recommendation performance. We fix the fraction of connected items by the spammers as 50% and plot the dependence of recommendation accuracy on the number ratio of spammers in Fig 2(c) and 2(d). In these two subfigures, the horizontal axis is the ratio of spammers added to the original network, the vertical axis is recommendation precision. The number of edges carried by each spammer is set to 10, 20, 30, 40 and 50 respectively. As we can see from the figure, with the increase of the edges carried by spammers, precision decreases increasingly faster. At the same time, we find that decreasing rate of precision in CF is smaller than MD, which implies that the robustness of CF algorithm to spammers is higher than MD.

**Fig 3:**
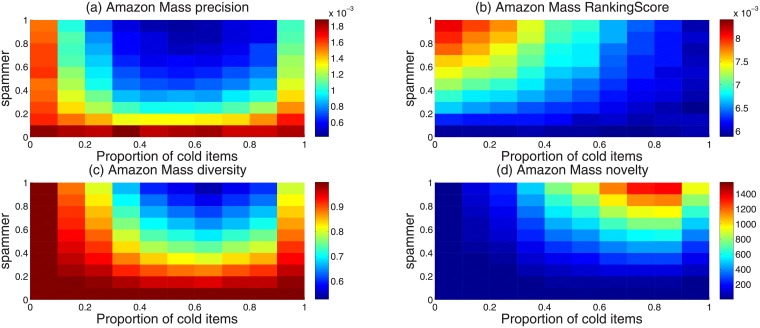
(Color online) The heatmap of four recommendation metrics in the parameter space of spammers ratio and cold item ratio. The recommendation metrics include (a) precision, (b) ranking score, (c) diversity and (d) novelty.

To understand the influence of spammers on recommendation performance more deeply, we consider all the four recommendation metrics introduced above and study their values in the parameter space of spammer ratio and cold item ratio. In simulation, we fixed the number of edges of each spammer as the average user degree of the real network. The heatmaps of ranking score, precision, diversity and novelty are shown in Fig 3. The horizontal axis is the ratio of spammers’ links connected to cold items. When the ratio is 0, all edges are connected to cold items. When the ratio is 1, all edges are connected to items randomly. The vertical axis is the ratio of spammers in the network. As we can see in Fig 3, when spammers ratio is large, recommendation diversity is maximum when the ratio of cold items is at about 20%. The is because a lot of spammers successfully push some original cold items into the recommendation list. As the cold items are different in each user’s recommendation list, the hamming distance between users’ recommendation list become larger, resulting in a high recommendation diversity.

Similarly, when the ratio of cold items is about 20%, novelty is relatively low. In this case, a lot of niche items are pushed into the recommendation list. These niche items reduce the average degree of the recommendation items, leading to a lower novelty. It can also be found when the proportion of cold item is around 20%, the value of ranking score is relatively large while the value of precision is relatively low. This is because the cold items that are pushed up into the recommendation list are not the probe set items liked by the users. The recommendation accuracy thus becomes lower.

The previous research results show that the accuracy of recommendation algorithm is affected by the entry of spammers into the network. In order to withstand the effects of spammers, a possible solution is to use the KNN approach. For example, in the Mass Diffusion algorithm, in the diffusion process of resource from users to items, we sort the resource values obtained by all users, only considering *α* fraction of users (*α* = 0.01, 0.02, …, 1) with highest resource and diffuse their resource back to the item side. The KNN apporach can to some degree withstand the interference of spammers. This is because most spammers are not within the top *α* users with the highest diffusion resource (highest similarity) to the target user. The KNN approach improve the recommendation accuracy by eliminating these spammers. The effectiveness of KNN approach indicates that the key to improving the robustness of recommendation algorithms is to develop a way to identify spammers and eliminate their contribution in computing recommendation score for items.

In general, real users have relative stable preference when selecting items, so the similarity between the selected items by real users is usually high. However, the spammer purchasing behaviors are assigned by the Internet water army company. Therefore, the similarity among the items purchased by spammers tend to be low. Based on the above assumption, we design the following algorithm based on KNN apporach to improve the robustness of recommendation algorithms. First, we calculate the average similarity between all the items selected by a user, and denote it as a vector *ω*. The vector *ω* will be used to improve the KNN approach. Instead of adopting the *γ* fraction of users with highest diffusion score *f*, the improved KNN approach rank users with *f* * *ω*^*θ*^. Here, *θ* adjusts the weight of vector *ω* in the KNN algorithm. When *θ* = 0, the method returns to the original KNN method. When *θ* > 0, the KNN approach only consider the contribution of the users who are not only most similar to the target user (as their received diffusion score is high), but also have stable preference in choosing items (high similarity between his/her own selected items). Here, we tested several value of *θ*, i.e. 0, 0.5, 1 and 2.

**Fig 4:**
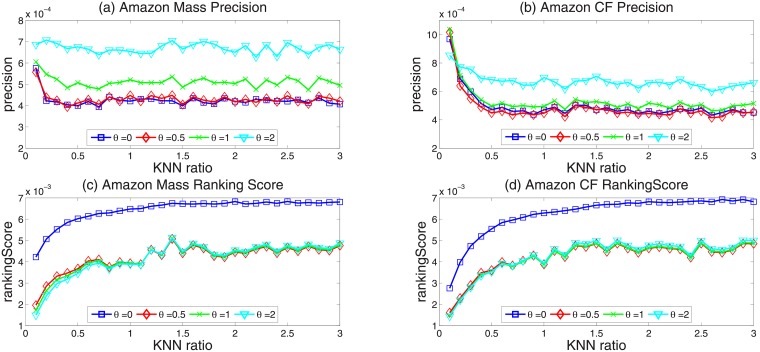
(Color online) the Precision and RankingSocre of improved KNN approach with different parameter *θ*.

**Fig 5:**
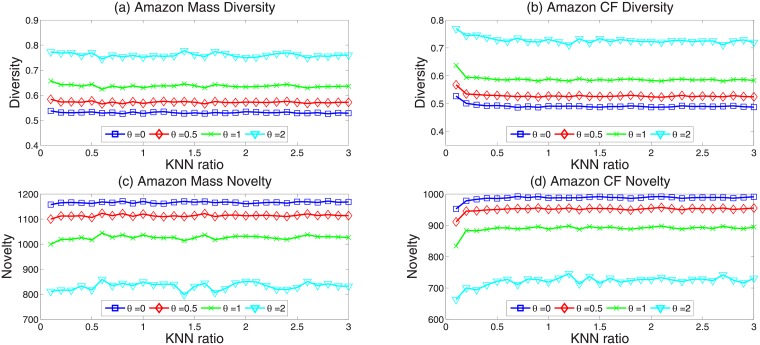
(Color online) the diversity and novelty of improved KNN approach with different parameter *θ*.

We respectively calculate precision, ranking score, diversity and novelty in the case of *θ* = 0, 0.5, 1, 2 with the improved KNN approach. We found that for the improved KNN approach, when *θ* = 2, the proposed algorithm can significantly improve both the diversity and the recommendation accuracy in comparison with the original KNN approach. In Figs 4 and 5, the horizontal axis is KNN ratio *γ* from 0.01 to 0.3. Specifically, Fig 4 (a) and 4(b) show the results of precision, Fig 4(c) and 4(d) show the results of ranking score. Fig 5(a) and 5(b) show the results of diversity, Fig 5(c) and 5(d) show the results of novelty. Obviously, when *θ* = 2, the new algorithm performs much better than the original KNN approach, both in the recommendation accuracy and recommendation diversity.

## Discussion

In this paper, we systematically study the effect of different spamming behavior on recommender systems. We found that not all type of the spamming behavior will significantly mislead the recommender systems. Only when some edges of the spamming users are connected to hot or niche items and the rest edges are connected to items randomly, will recommender systems be largely misguided. We further enhance the robustness of existing recommendation algorithms against the spamming users by an improved KNN approach. The improved algorithm leads to a remarkable improvement in both recommendation accuracy and diversity.

In terms of recommendation algorithms, we employ two recommendation methods, i.e. the mass diffusion method and collaborative filtering method. These two recommendation methods are actually very representative. The mass diffusion method is a highly-cited recommendation method developed by physicists. The collaborative filtering method is a widely-applied recommendation method developed by computer scientists. We compare the capability of traditional KNN approach and an improved KNN approach by us for enhancing the robustness of these two representative recommendation methods. Note that, the improved KNN approach is actually very general and can be applied to any user-similarity-based recommendation algorithms. For the moment, our algorithm mainly works for the recommendation algorithms based on user-similarity. It will be an interesting and useful extension to incorporate our algorithm to other types of recommendation methods such as the matrix factorization.

Based on similar principle, some other methods to enhance the recommendation robustness can be designed. For example, one can directly delete the users with relatively low internal similarity in the data, and obtain a higher recommendation accuracy when applying recommendation algorithms on these “cleaner” data. In addition, our method can also be applied to detect users and relationships who/which are spamming and redundant in different systems [48, 49]. This can help the commercial system to identify the Internet water army users. Finally, we believe our method can be further improved if purchase time information is incorporated in the method. For example, if some users suddenly push some niche items and then have no activity for a long period of time, they are more likely to be spammers.

## Supporting information

S1 File Supplementary information of enhancing the robustness of recommender systems against spammers. (PDF)

S1 Fig (a), (b) show the relationship between recommendation accuracy and the ratio of the number of links to cold items and the number of total links. (c), (d) show the relationship between recommendation accuracy and the number of links of every false user.
(EPS)

S2 Fig (a), (b) show the relationship between recommendation accuracy and the ratio of the number of links to cold items and the number of total links. (c), (d) show the relationship between recommendation accuracy and the number of links of every false user.
(EPS)

S3 Fig The heatmap of four recommendation metrics in the parameter space of spammers ratio and cold item ratio. The recommendation metrics include (a) precision, (b) ranking score, (c) diversity and (d) novelty.
(EPS)

S4 Fig The heatmap of four recommendation metrics in the parameter space of spammers ratio and cold item ratio. The recommendation metrics include (a) precision, (b) ranking score, (c) diversity and (d) novelty.
(EPS)

S5 Fig The Precision and RankingSocre of improved KNN apporach with different *θ*. (EPS)

S6 Fig The Precision and RankingSocre of improved KNN apporach with different *θ*. (EPS)

S7 Fig The diversity and novelty of improved KNN approach with different parameter *θ*. (EPS)

S8 Fig The diversity and novelty of improved KNN approach with different parameter *θ*. (EPS)
