# Supplementary material for: Enhancing the robustness of recommender systems against spammers
Source: PLoS One. 2018 Nov 1;13(11):e0206458. doi: 10.1371/journal.pone.0206458 (PMC6211683; doi:10.1371/journal.pone.0206458)
Supplement: S1 File — (PDF) [file pone.0206458.s001.pdf]

Supplementary Information

Enhancing the robustness of recommender systems against spammers

Chengjun Zhang, Jin Liu, Yanzhen Qu, Tianqi Han, Xujun Ge and An Zeng

I. RECOMMENDATION ACCURACY IN DELICIOUS AND RYM WITH DIFFERENT SPAMMER RATIOS.

First, we investigate how the fraction of connected cold items and fraction of randomly selected items influence the recommendation accuracy. As shown in S1(a)(b) and S2(a)(b) Figs, the horizontal axis is the fraction of links connected to cold items (the remaining links are randomly linked to items), and the vertical axis represents recommendation accuracy measured by precision. It can be easily observed that when the fraction of cold items in the total edges is around 20%, the recommendation accuracy is affected most significantly.

Meanwhile, in order to study how the number of spammers affects the recommendation performance in Delicious and RYM data. We fix the fraction of connected items by the spammers as 50% and plot the dependence of recommendation accuracy on the number ratio of spammers in S1(c)(d) and S2(c)(d) Figs. In these subfigures, the horizontal axis is the ratio of spammers added to the original network, the vertical axis is recommendation precision. The number of edges carried by each spammer is set to 10, 20, 30, 40 and 50 respectively. As we can see from the figure, with the increase of the edges carried by spammers, precision decreases increasingly faster. At the same time, we find that decreasing rate of precision in CF is smaller than MD, which implies that the robustness of CF algorithm to spammers is higher than MD.

Obviously, both figures show consistent results as S2 Fig in the paper.

II. THE HEATMAP OF THE RECOMMENDATION METRICS UNDER DIFFERERNT SPAMMING PARAMETERS IN DELICIOUS AND RYM.

To understand the influence of spammers on recommendation performance more deeply, we fixed the number of edges of each spammer as the average user degree of the real network. The heatmaps of ranking score, precision, diversity and novelty are shown in S3 and S4 Figs. The horizontal axis is the ratio of spammers' links connected to cold items. When the ratio

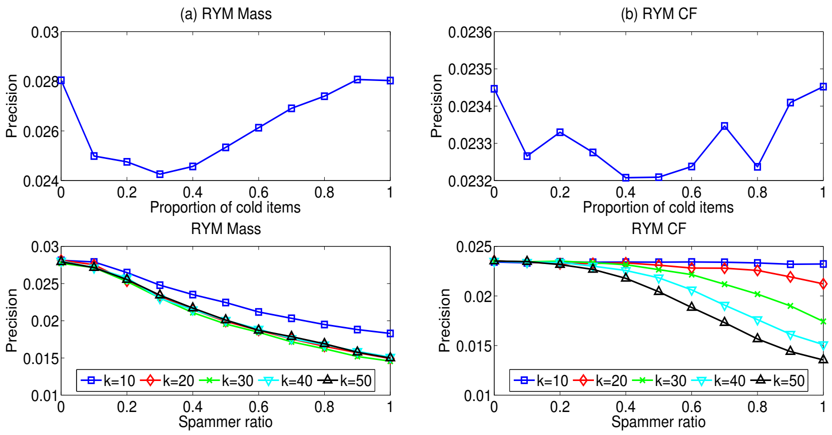

S1 Fig. (a), (b) show the relationship between recommendation accuracy and the ratio of the number of links to cold items and the number of total links. (c), (d) show the relationship between recommendation accuracy and the number of links of every false user.

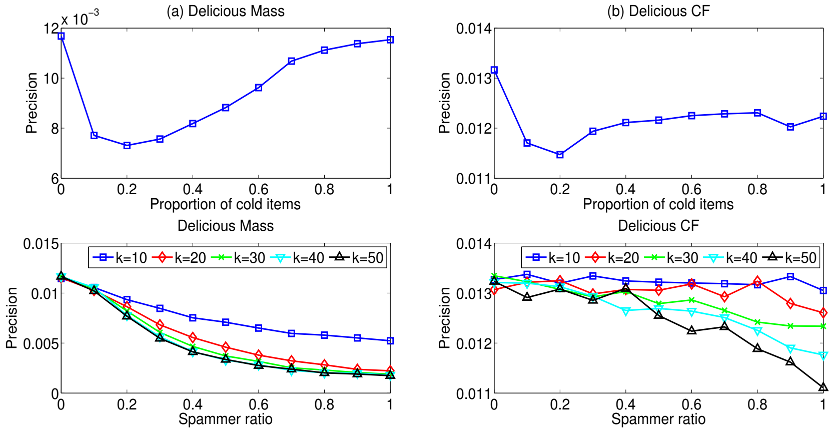

S2 Fig. (a), (b) show the relationship between recommendation accuracy and the ratio of the number of links to cold items and the number of total links. (c), (d) show the relationship between recommendation accuracy and the number of links of every false user.

is 0, all edges are connected to cold items. When the ratio is 1, all edges are connected to items randomly. The vertical axis is the ratio of spammers in the network. As we can see in S3 and S4 Figs, when spammers ratio is large, recommendation diversity is maximum when the ratio of cold items is at about 20%. The is because a lot of spammers successfully push some original cold items into the recommendation list. As the cold items are different in each user's recommendation list, the hamming distance between users' recommendation list become larger, resulting in a high recommendation diversity.

Similarly, when the ratio of cold items is about 20%, novelty is relatively low. In this case, a lot of niche items are pushed into the recommendation list. These niche items reduce the average degree of the recommendation items, leading to a lower novelty. It can also be found when the proportion of cold item is around 20%, the value of ranking score is relatively large while the value of precision is relatively low. This is because the cold items that are pushed up into the recommendation list are not the probe set items liked by the users. The recommendation accuracy thus becomes lower.

Obviously, the four metrics show similar trends to S3 Fig in the paper.

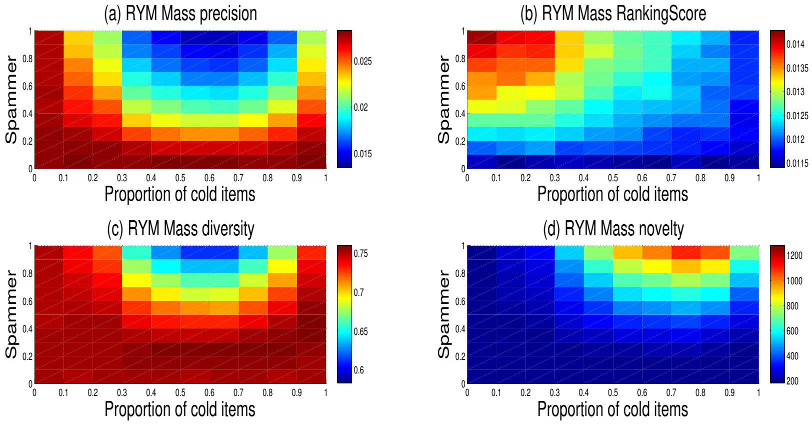

S3 Fig. The heatmap of four recommendation metrics in the parameter space of spammers ratio and cold item ratio. The recommendation metrics include (a) precision, (b) ranking score, (c) diversity and (d) novelty.

III. RECOMMENDATION METRICS OF THE IMPROVED KNN APPROACH IN DELICIOUS AND RYM DATA.

In order to study systematically the performance of the improved KNN approach in precision, ranking score, diversity and novelty in Delicious and RYM data. We respectively calculate precision, ranking score, diversity and novelty in the case of θ=0, 0.5, 1, 2 with the improved KNN approach. We found that for the improved KNN approach, when $\theta = 2$, the proposed algorithm can significantly improve both the diversity and the recommendation accuracy in comparison with the original KNN approach. In S5, S6, S7 and S8 Figs, the horizontal axis is KNN ratio α from 0.01 to 0.3. Specifically, S5(a)(b) and S6(a)(b) Figs show the results of precision in RYM and Delicious respectively. S5(c)(d) and S6(c)(d) Figs

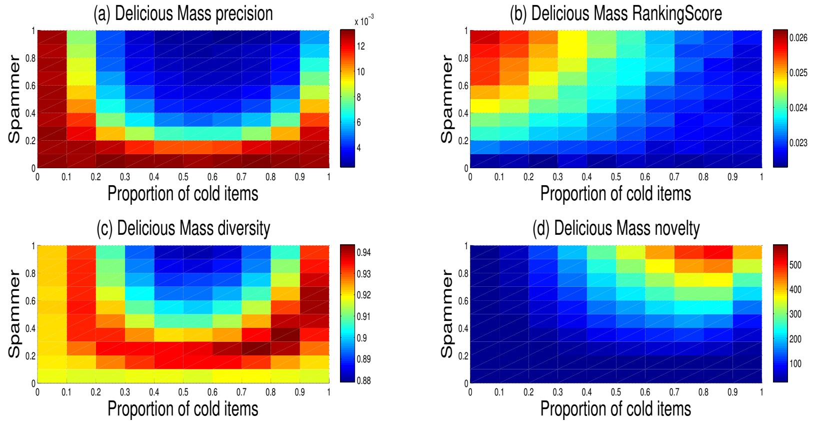

S4 Fig. The heatmap of four recommendation metrics in the parameter space of spammers ratio and cold item ratio. The recommendation metrics include (a) precision, (b) ranking score, (c) diversity and (d) novelty.

show the results of ranking score. S7(a)(b) and S8(a)(b) Figs show the results of diversity in RYM and Delicious respectively, S7(c)(d) and S8(c)(d) Figs show the results of novelty. Obviously, when $\theta = 2$, the new algorithm performs much better than the original KNN approach, both in the recommendation accuracy and recommendation diversity. The results of RYM and Delicious also show the same trends as S4 and S5 Figs in the paper.

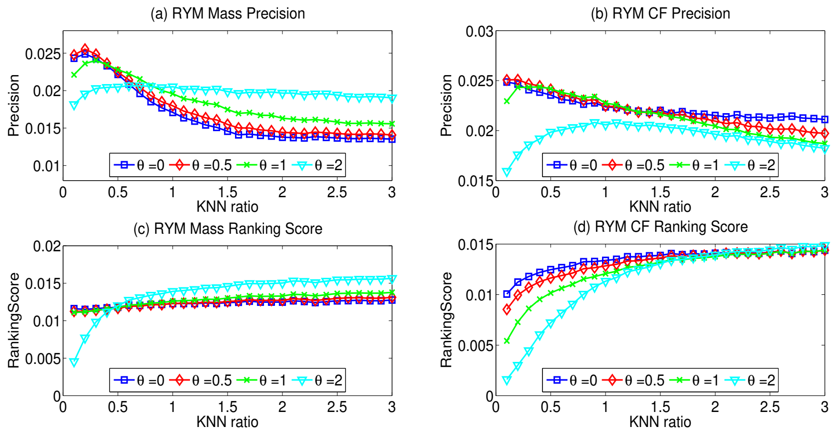

S5 Fig. the Precision and RankingSocre of improved KNN apporach with different θ.

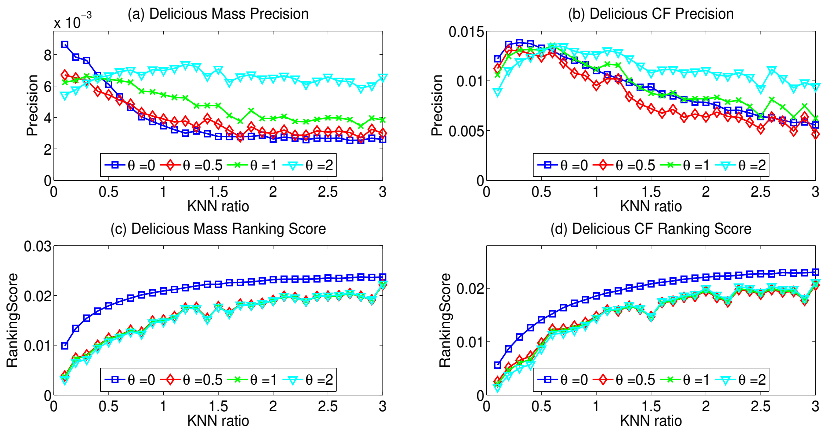

S6 Fig the Precision and RankingSocre of improved KNN apporach with different θ.

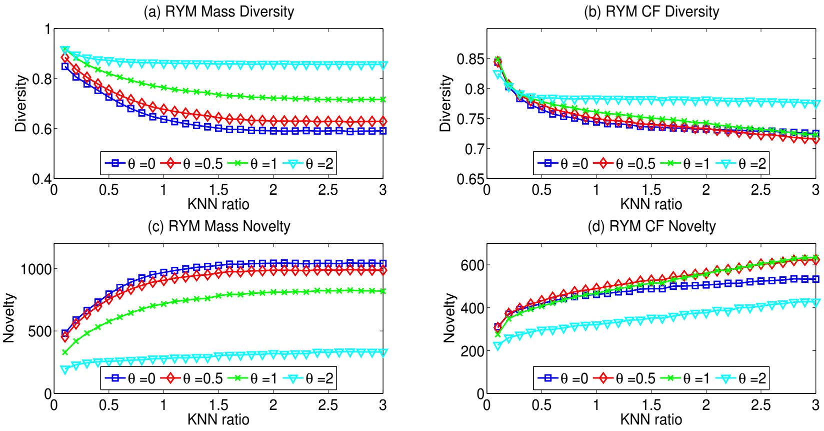

S7 Fig. the Diversity and Novelty of improved KNN approach with different parameter θ.

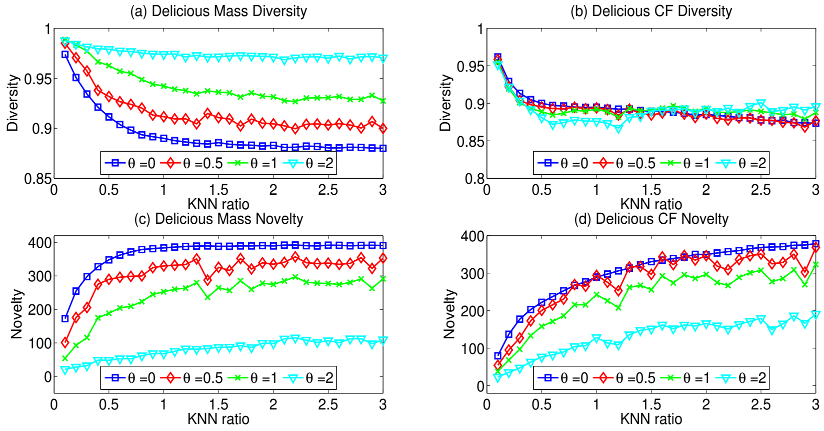

S8 Fig. the Diversity and Novelty of improved KNN approach with different parameter θ.
